# EBP1: A crucial growth regulator downstream of receptor kinases across kingdoms

**DOI:** 10.1371/journal.pbio.3000056

**Published:** 2018-11-07

**Authors:** Martin Stegmann

**Affiliations:** Phytopathology, School of Life Sciences Weihenstephan, Technical University of Munich, Freising, Germany

## Abstract

Controlling organ growth and development is crucial for all multicellular organisms and is controlled by plasma membrane localized receptor kinases (RKs) across kingdoms, including animals and plants. A central RK in plants is FERONIA (FER), which perceives endogenous rapid alkalinization factor (RALF) peptides to regulate a plethora of biological responses, including growth and development. However, it remained largely unknown how RALF sensing by FER at the plasma membrane is translated into a nuclear response. A key step forward is presented by Li and colleagues, who show that FER increases *ERBB3 binding protein* 1 (*EBP1*) mRNA translation and directly phosphorylates EBP1 to shift its subcellular localization from the cytoplasm to the nucleus where it controls growth and development through its regulation of transcription. Importantly, EBP1 is described as a transcriptional and translational regulator in mammals by acting downstream of epidermal growth factor receptor (EGFR) signaling, suggesting that animals and plants use similar conserved pathways to fine-tune growth and development. Furthermore, this work highlights the importance of protein translation as a direct output of RK signaling, a mechanism that is largely unknown in plants.

## Abstract 2

This Primer discusses the recent demonstration that EBP1 is a major transcriptional and translational regulator across kingdoms, acting downstream of EGFR signalling in animals and the central receptor kinase FERONIA in plants.

Controlling growth and development in a spatiotemporal manner is crucial for all multicellular organisms. Misregulation of the underlying signaling pathways is often associated with excessive tissue proliferation, leading to oncogenesis in animals and humans. Similarly, organ development and size control are important in plants, and understanding the underlying mechanisms may aid future biotechnological approaches to increase crop yield.

An important family of proteins regulating growth and development are plasma membrane-localized receptor kinases (RKs), which represent a major family of signal-transducing proteins conserved across kingdoms [1]. Their function is mainly linked to their ability to bind extracellular ligands, such as peptides or other small molecules, with subsequent transmembrane activation of downstream signaling pathways, resulting in an appropriate cellular output.

In recent years, RKs belonging to the *Catharanthus roseus* receptor-like kinase 1-like (CrRLK1L) family emerged as important regulators of plant reproduction, responses to the environment, growth, and development [2]. In *Arabidopsis*, the first discovered and best described CrRLK1L is FERONIA (FER), which was initially found to be required for pollen tube perception in the female reproductive organ, the pistil, and later found to be involved in a plethora of responses, including root hair growth, cell elongation, hormone responses, and plant immunity [2]. Yet for a long time, it was unclear how FER is activated, as no ligands were described. Recent research has shown that FER (and related CrRLK1Ls) perceive peptide ligands belonging to the family of rapid alkalinization factors (RALFs) [3–7]. There are at least 36 genes encoding for RALF peptides in the genome of *Arabidopsis thaliana*, raising the possibility that FER and related RKs fulfil a multitude of functions by perceiving tissue- and/or context-specific RALF peptides, some of which also modulate FER and related proteins in an antagonistic manner [4,5]. The first described FER ligand was the root-specific RALF1 [3], and the last years have provided significant insights into the signal transduction pathways activated in response to its perception. The small glycosylphosphatidylinositol (GPI)-anchored proteins LORELEI (LRE) and the related LRE-like GPI-anchored protein 1 (LLG1) are required for FER plasma membrane localization in a tissue-specific manner and are discussed as potential RALF coreceptor proteins [8]. The receptor-like cytoplasmic kinase (RLCK) RPM1-interacting protein kinase (RIPK) and FER mutually phosphorylate each other to regulate cell growth in roots [9]. Furthermore, FER was shown to activate rho of plants (ROP) signaling to positively regulate auxin-dependent root hair growth, which in turn is counteracted by abscisic acid (ABA)-induced FER phosphorylation via inhibition of the A-type protein phosphatase 2C (PP2C) ABA insensitive 2 (ABI2) [10–12]. Despite the advances in our understanding of early FER-dependent responses, it remains largely unknown how this central regulatory RK regulates downstream cellular responses beyond the plasma membrane, including transcriptional or translational reprogramming.

In this issue of *PLOS Biology*, Li and colleagues [13] identify the plant ortholog of the mammalian DNA/RNA-binding protein ERBB3 binding protein 1 (EBP1) as an interactor of FER and potentially other CrRLK1L family members (Fig 1A and 1B). The authors show that *EBP1* mRNA translation (not transcription) is enhanced after RALF1 perception in a FER-dependent manner, resulting in elevated EBP1 protein levels (Fig 1A). Subsequently, EBP1 is translocated to the nucleus, a response that is dependent on FER-mediated phosphorylation (Fig 1B). Here, EBP1 associates with RALF1 responsive genes and regulates their transcription. Genetically, *EBP1* largely serves as a negative regulator of RALF1 signaling.

**Fig 1:**
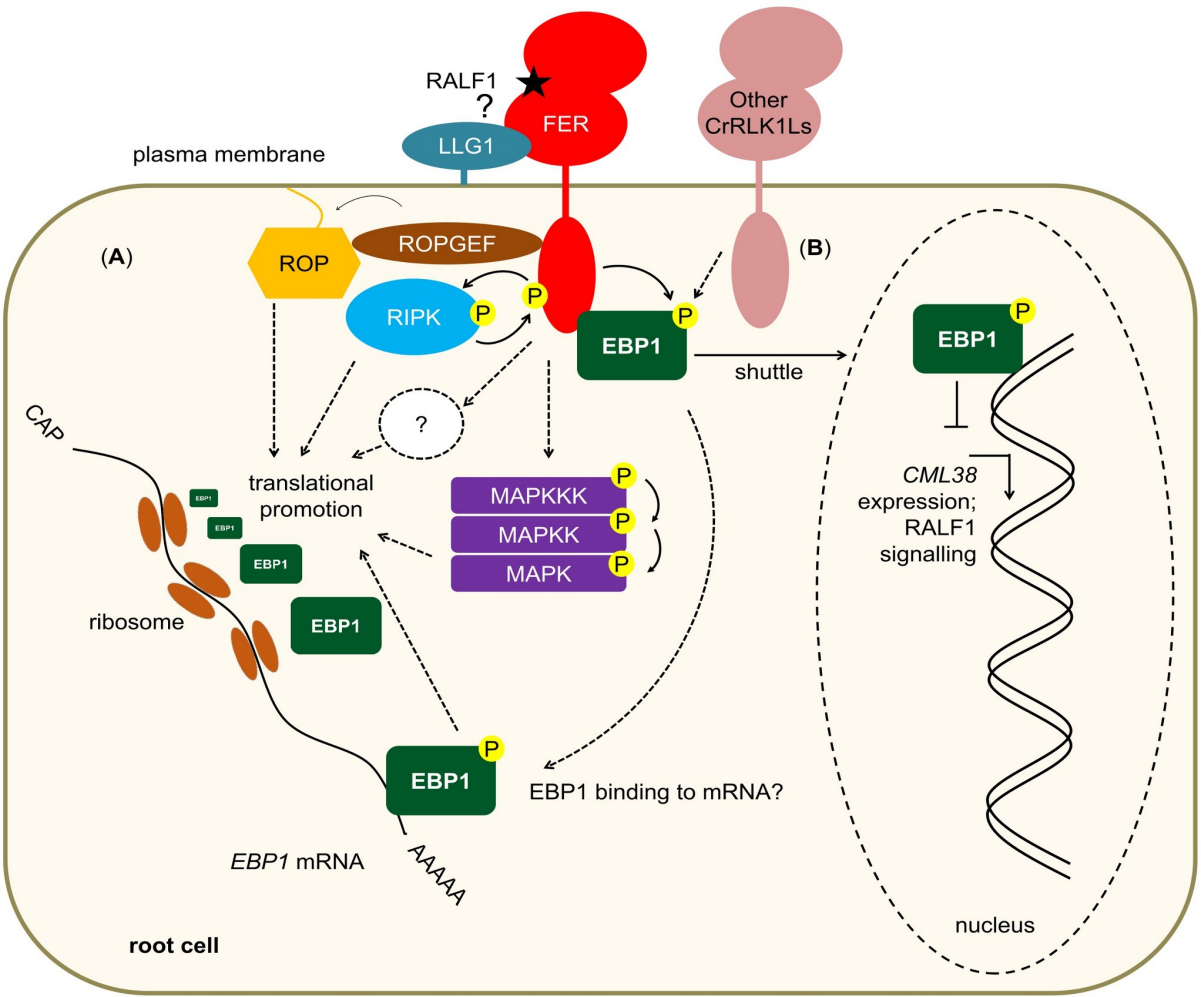
(A) RALF1 perception by FER results in the activation of downstream pathways, including RIPK, ROPGEF–ROP, and MAPK activation. Also, FER signaling results in the translational up-regulation of EBP1 protein levels via yet unknown mechanisms. (B) RALF1 perception leads to FER phosphorylating the FER-associated EBP1, which subsequently is shuttled to the nucleus where it binds to RALF1 responsive genes and regulates their expression to inhibit RALF1 signaling, e.g., transcriptional down-regulation of *CML38*. Dashed lines with arrowheads: possible suggested mechanisms/connections but no direct experimental evidence yet. *CML38*, *CALMODULIN-like protein 38*; EBP1, ERBB3 binding protein 1; FER, FERONIA; MAPK, mitogen-activated protein kinases; P, phosphorylation; RALF, rapid alkalinization factor; RIPK, RPM1-interacting protein kinase; ROP, rho of plants.

The present study shows a direct link from the perception of a peptide signal by an RK to downstream nuclear responses, including transcriptional reprogramming and enhancement of translation, something that is largely unknown in plants. The geminivirus nuclear shuttle protein (NSP)-interacting kinase 1 (NIK1) RK associates with ribosomal protein L10A (RPL10A) to redirect the protein to the nucleus, with subsequent repression of translational machinery subunits [14]. Furthermore, upon relocalization, RPL10 interacts with the MYB-like protein L10-interacting myb domain-containing protein (LIMYB) to suppress the expression of translational machinery genes, which results in enhanced tolerance to begomovirus infection [15]. Interestingly, overexpression of *NIK1* in tomatoes results in stunted growth [14], suggesting that RK-induced translational regulation is a common mechanism controlling growth and development. Yet the extracellular signal resulting in NIK1-triggered RPL10A nuclear relocalization remains to be discovered. Therefore, the study by Li and colleagues is the first example showing a direct link from ligand perception by an RK to subsequent translational regulation during growth and development, yet the underlying mechanism remains to be elucidated. Interestingly, translational regulation was also recently shown to be crucial for the activation of plant immunity triggered by the perception of bacterial elongation factor thermo unstable (EF-TU) by EF-TU receptor (EFR) [16], suggesting that it represents a widespread mechanism downstream of RK signaling. Furthermore, it highlights the importance of mRNA-related processes (e.g., translational regulation) for signal transduction after perception of an extracellular cue, something that is poorly understood in plants.

Another intriguing finding by Li and colleagues is that FER, upon enhancing its translation, directly phosphorylates the cytoplasmic nuclear shuttle protein EBP1 to control gene expression. This is the first example for such a “short-cut” signaling pathway avoiding canonical step-wise phosphorylation cascades for the activation of a downstream transcriptional response in plants. A similar mechanism from ligand perception to transcriptional responses has recently been suggested for pathogen-associated molecular pattern (PAMP)-triggered immune signaling. Perception of ET-TU results in the activation of the EFR receptor complex-associated RLCK *botrytis*-induced kinase 1 (BIK1), which is crucial for signal transduction during non-self–perception [17]. A recent paper has shown that BIK1 also localizes to the nucleus, suggesting that it can simultaneously activate early signaling events (e.g., PAMP-triggered apoplastic reactive oxygen species production and influx of calcium ions at the plasma membrane) as well as later responses, such as transcriptional reprogramming of the cell when in the nucleus [18]. Importantly, though, a receptor-induced relocalization of BIK1 from the plasma membrane to the nucleus, as shown for FER–EBP1, remains to be tested.

A similar regulation of RK signaling is well known from the mammalian field, likewise regulating tissue growth and development. An important family of receptor kinases in mammals are the ERBB receptor tyrosine kinases with epidermal growth factor receptor (EGFR)/ERBB-1 being the most prominent and best studied member [19]. EGFR perceives EGF peptide ligands and is essential for ductal development in the mammary gland. Overdosed EGFR signaling is associated with cancer development in various tissues [20]. EGFR signaling was shown to be regulated by EBP1, which is similarly recruited to the nucleus after EGF perception and subsequently regulates the expression of EGF-dependent genes [21]. EBP1 was also shown to bind to RNA directly as part of ribonucleoprotein (RNP) complexes and regulate translation during EGF–EGFR signaling [22,23]. Thus, an intriguing finding of the work by Li and colleagues [13] is that EBP1 function is conserved across kingdoms and likewise regulates growth and organ development in both mammals and plants. Furthermore, phylogenetic analysis shows that EBP1 orthologues can be found in all eukaryotic kingdoms of life (including plants, animals, fungi, and protista), raising the possibility that EBP1 function represents an ancient signaling mechanism to regulate growth and development. Interestingly, the study by Li and colleagues shows for the first time that *EBP1* mRNA translation is regulated in response to the perception of an upstream peptide (as shown for RALF1), which represents a novel regulatory mechanism of EBP1 activity first described in plants. The current study also suggests that additional CrRLK1Ls may interact with EBP1 in plants. This opens up the possibility that EBP1 may work as a central downstream hub for CrRLK1L members after perception of their respective ligands. Whether EBP1, similar to its function in mammals, can also be part of RNP complexes and to regulate translation remains to be addressed in future studies.

Despite a broad involvement of EBP1 in RALF–FER signaling, the mutants or overexpressors are unaffected in reproduction, the first described FER function. *FER* is expressed throughout most tissues, while its described signaling partners show tissue-specific expression patterns and belong to multigene families. For example, *LRE* is expressed in reproductive tissue and *lre* mutants show a similar reproductive defect as *fer*. By contrast, in vegetative tissue, the function of LRE is taken over by its closest homolog LLG1 [8]. In the case of *EBP1*, phylogenetic analysis shows that it is a single gene in *Arabidopsis*, raising the question whether reproductive tissue is an exception for EBP1-like regulation of FER signaling or whether yet uncharacterized proteins with low homology to EBP1 may take over a similar function in this context. Importantly, recent work has shown that some FER-regulated pathways are independent or partially independent of FER kinase activity, including fertilization [24,25]. This suggests that some responses may not require FER-dependent EBP1 phosphorylation, which could explain the lack of phenotype in plant reproduction reported for mutants affected in *EBP1*. Also, it remains to be explained how EBP1 and FER have largely overlapping but also opposite functions in some analyzed responses. FER and other CrRLK1Ls can have opposing functions, e.g., the closely related FER and ANXUR1 (ANX1) during immune regulation [4,26]. ANX1 is also a potential interactor of EBP1, raising the possibility that related RKs with opposing downstream output may differentially regulate EBP1. To further increase complexity, recent research has shown that FER and related RKs can perceive RALF peptides with antagonistic function perceived by the same individual RK [4,5]. Therefore, an intriguing hypothesis is that EBP1 serves as a downstream hub and is either activated or repressed via distinct FER-dependent phosphorylation sites to regulate subsequent responses. Interestingly, the authors show that steady-state protein levels of EBP1 are elevated in *fer* mutants. This may sound contradictory to the FER-dependent EBP1 protein stabilization but could be explained by FER (depending on the presence of an activating or repressing RALF peptide) having opposing functions on EBP1 stability and thus fine-tuning downstream responses. Also, future studies need to address the mechanistic basis of translational repression/activation during RK signaling in plants, and FER–EBP1 may serve as a crucial example. It will be important to test whether known downstream FER signaling components, such as RIPK or ROPs, may be involved in this process or whether yet uncharacterized proteins are responsible (Fig 1A). Interestingly, ROP2, a known downstream FER signaling protein [10], was shown to activate target of rapamycin (TOR) kinase signaling in response to auxin perception to regulate translation in plants [27], resembling a similar mechanism in animals [28]. Given the function of EBP1 as part of RNP complexes in mammals, an intriguing possibility would also be that EBP1, upon phosphorylation by FER, may regulate its own translation as part of a feed forward loop (Fig 1A). FER signaling also includes the activation of mitogen-activated protein kinases (MAPKs) [29], which were suggested to be involved in translational reprogramming of cells upon EF-TU perception [16] and might thus likewise be involved in FER signaling. Also, it will be interesting to test how widespread translational regulation is during FER and RK signaling in general, opening up new avenues for our understanding of the mechanistic basis of RK-mediated signal transduction and closing gaps between the plasma membrane and the inner life of the plant cell.

## Abbreviations

ABA: abscisic acid
ABI2: ABA insensitive 2
ANX1: ANXUR1
BIK1: *Botrytis*-induced kinase 1
CrRLK1L: *Catharanthus roseus* receptor-like kinase 1-like
EF-TU: elongation factor thermo unstable
EFR: EF-TU receptor
EGFR: epidermal growth factor receptor
EBP1: ERBB3 binding protein 1
FER: FERONIA
GPI: glycosylphosphatidylinositol
LIMYB: L10-interacting myb domain-containing protein
LLG1: LRE-like GPI-anchored protein 1
LRE: LORELEI
MAPK: mitogen-activated protein kinase
NIK1: NSP-interacing kinase 1
PAMP: pathogen-associated molecular pattern
PP2C: protein phosphatase 2C
RALF: rapid alkalinisation factor
RIPK: RPM1-interacting protein kinase
RLCK: receptor-like cytoplasmic kinase
RNP: ribonucleoprotein
ROP: rho of plants
RK: receptor kinase
RPL10A: ribosomal protein L10A
TOR: target of rapamycin
